# A multicenter study of maternal conditions and amniotic fluid volume in Ethiopia in 2023

**DOI:** 10.14814/phy2.15994

**Published:** 2024-03-27

**Authors:** Besfat Berihun Erega, Wassie Yazie Ferede, Fillorenes Ayalew Sisay, Abeba Belay Ayalew, Rahel Birhanu Arage

**Affiliations:** ^1^ Department of Midwifery, College of Medicine and Health Sciences Debre Tabor University Debre Tabor Ethiopia

**Keywords:** amniotic fluid, Ethiopia, maternal condition, oligohydramnios, polyhydramnios

## Abstract

The amniotic fluid is a protective liquid found in amniotic found in the amniotic sac and mainly containing water and some solid substances including epitheloid and fibroblastic type cells. Most of the studies conducted about amniotic fluid volume (AFV) reported fetal and placental factors as a determinant of AFV. The aim of this study is to examine maternal and obstetric conditions in relation to AFV among women with term pregnancies. A multicenter institutional based cross‐sectional study was conducted among clients attending selected public hospitals of South Gondar Zone, Ethiopia from January 01, 2023 to May 30, 2023. The sample size was calculated by using the assumption of single population proportion formula considering the prevalence value of 50%, 95% confidence interval, and margin of error 5% and 10% non respondent rate. In our study rural residency AOR = 3.21 (1.19–5.37), chronic illness AOR = 2.12 (1.33–4.61), short inter pregnancy interval AOR = 3.03 (2.18–6.28), Hypermesis gravidarum AOR = 1.19 (1.02–4.41), and maternal diabetics AOR = 2.16 (1.32–4.75) had significant association with the outcome variable. These maternal conditions may be correlated with an abnormal volume of amniotic fluid.

## 1 INTRODUCTION

The amniotic fluid is a protective liquid found in amniotic sac that mainly contains water and some other solid substances like epitheloid and fibroblastic type cells. The Amniotic Fluid Index (AFI) is a standardized way to assess the sufficiency of amniotic fluid quantity in pregnancy (Bakhsh et al., 2021; Ray et al., 2017). Most of the solid substances within the amniotic fluid are indicators of the fetal well‐being and the amniotic fluid helps the fetus by providing temperature stability, cushioning and a necessary presence in collapsed airways to help stimulate lung development (Raghuwanshi & Aggarwal, 2017). Amniotic fluid swallowed by the fetus helps in the formation of the gastrointestinal tract and promotes musculoskeletal development and prevents the fetus from mechanical jerks and shocks (Ray et al., 2017). The amniotic fluid is believed to have its origin as a simple exudation from the tissues of the fetus. After the formation of the placenta, however, an exudation of lymph takes place from the maternal vessels. Later it becomes mixed with fetal urine and, in common with urine and blood, contains proteins, urea and salts, in addition to water (Metzger et al., 1990; Tong et al., 2009).

Amniotic fluid volume (AFV) does not change significantly from day to day, but generally it increases with the growth of fetus reaching a peak at 34 weeks of gestation (over 800 mL) (Raghuwanshi & Aggarwal, 2017; Hashimoto et al., 1993).The AFV varies with the gestational age from 200 mL at 16 weeks, 1000 mL at 28 weeks, 900 mL at 36 weeks and 800 mL at 40 weeks of gestation term pregnancy (Moore, 2010; Raghuwanshi & Aggarwal, 2017). Despite the difficulty in correlating the two parameters (AFI and AFV), a phenomena with AFI of greater than 24 cm is known as Polyhydramnios (Bakhsh et al., 2021). the determinant factors for Polyhydramnios include maternal diabetes mellitus (Mathew et al., 2008), higher gestational age (Mathew et al., 2008; Vanda et al., 2022) fetal heart failure, abnormal swallowing, multiparity (Morris et al., 2014; Vanda et al., 2022) and congenital infection (Bakhsh et al., 2021). On the other side, AFI of the amniotic volume less than 5 cm is referred as oligohydramnios (Bakhsh et al., 2021; Fida et al., 2007). The common determinants for oligohydramnios include premature rupture of membranes, intrauterine growth restriction and birth defects, pregnancy induced hypertension, Anemia and primiparity (Bakhsh et al., 2021; Mathew et al., 2008).

Most of the studies conducted about AFV reported fetal and placental factors as a determinant of AFV (Madhavi & Rao, 2015; Timur et al., 2018). The commonly fetal risk factors in determining AFV are dependent on maternal preconception and pregnancy conditions. Hence, the goal of this study was to examine maternal and obstetric conditions in relation to AFV among women with term pregnancies.

## 2 METHODS AND MATERIALS

### 2.1 Study design and setting

A multicenter institutional based cross‐sectional study was conducted among clients attending selected public hospitals of South Gondar Zone (SGZ), Ethiopia from January 1, 2023 to May 30. 2023. SGZ is the one among the 10 administrative zones in Amhara region, Ethiopia. The town is found about 669 km northwest of Addis Ababa, the capital city of Ethiopia, and 97 km southwest of BahirDar, the capital city of the Amhara region and it has an elevation of 2706 m above sea level. Addis Zemen Hospital, Debre Tabor Hospital, Mekaneyesus Hospital and Simada Hospital were selected randomly among the eight public hospitals of SGZ.

### 2.2 Source population

All pregnant women who came for ANC service at term pregnancy in public hospitals of SGZ.

### 2.3 Study population

All pregnant women who came for ANC service at term pregnancy in selected public hospitals of SGZ during the study period.

### 2.4 Inclusion criteria

Women at gestational age of between 37 and 42 weeks.

### 2.5 Exclusion criteria

‐ Women with gestational age of less than 37 weeks and greater than 42 weeks.

‐ Multiple pregnancy.

‐ Premature rupture of membrane.

### 2.6 Dependent variable

AFV.

### 2.7 Independent variables

Maternal conditions, Sociodemographic characteristics and RH and obstetric conditions.

### 2.8 Sample size determination and sampling procedure

The sample size was calculated by using the assumption of single population proportion formula considering the prevalence value of 50%, 95% confidence interval (CI), margin of error 5% and 10% non respondent rate. Then the final sample size was calculated to be 384. To reach for the study participants systematic random sampling was employed after searching for the case flow of each study hospitals. Finally, the sampling interval was determined by dividing 4 months case report to sample size in each hospitals and the final k^th^ value for Addis Zemen Hospital, Debre Tabor Hospital, Simada Hospital and Mekaneyesus Hospital was 3.9, 4.4, 4.3, and 4.2 respectively. Hence the first case to come was taken as participant one and every four cases were selected.

### 2.9 Data collection procedures

Written informed consent was taken from all study participants for data collection, publication and dissemination process as per the declaration of Helsinki. The data was collected by four BSc degree holder midwives, using structured questionnaires after training was given for a day in each hospital. The questionnaire was prepared originally in English and was then translated into local language, Amharic for the purpose of data collection. It was translated back to English again for consistency and accuracy by language experts. Pretest was also done in three primary hospitals other than the study areas.

### 2.10 Ethical approval

The ethical clearance was obtained from the Institutional Ethical Review Board of the Debre Tabor University (reference number 00765/2023). Letter of permission was obtained from the clinical coordinator of each study hospital. Clear explanation about the purpose of the study was given along with the letter of support for all concerned body. Finally, written Informed Consent of the respondents was obtained after thoroughly explaining the aim of the study to each respondent. In addition all methods were performed in accordance with the relevant guidelines and regulations. Permission to publish the data analysis is also covered by Institutional ethical Review Board of the Debre Tabor University.

### 2.11 Data entry and analysis

After manually checking its completeness and consistency, the data were entered using Epi‐data version 4.6 software and analyzed using SPSS version 23 software. Then to know the crude association between AFV and determinant factors crude odds ratio (COR) was calculated with 95% CI. Variables with an odds ratio of ≤0.2 were considered for multivariate analysis. Variables with adjusted odds ratio of ≤0.05 were considered to determine the significance of association. Hosmer–Lemeshow goodness‐of‐fit test was used to check the model fitness, Poor fit was considered by a value less than 0.05.It was considered to have multi collinearity when VIF is greater than 10.

### 2.12 Outcome measures

After selection of the study participants, Participants in the study were given an ultrasound scan to measure AFI or SDP. The normality range of the ultrasound findings was defined as per the scientific measurements (normal AFI 5–25). Then we interviewed with the women for possible confounders of amniotic fluid abnormality.

## 3 RESULTS

### 3.1 Distribution of sociodemographic characteristics

A total of 384 respondents were included in this study with a response rate of 100%. majority of the respondents were in the age group of 30–49 years, orthodox Christian in religion, rural residents and monthly income greater than 2000ETB (Table 1).

Majority of our respondents have ANC followup (75.26%), were Multigravida (69.27%), and had no pregnancy related complications (Table 2).

In our study rural residency AOR = 3.21 (1.19–5.37), chronic illness AOR = 2.12 (1.33–4.61), short inter pregnancy interval AOR = 3.03 (2.18–6.28), Hypermesis gravidarum AOR = 1.19 (1.02–4.41), and maternal diabetics AOR = 2.16 (1.32–4.75) had significant association with the outcome variable (Table 3).

**TABLE 1:** Distribution of socio‐demographic characteristics of the respondents with their amniotic fluid volume at public hospitals in south Gondar zone, North West Ethiopia, 2023 (*N* = 384).

Variables	Oligohydramnios	Normal AFV	Polyhydramnios	Total *N* (%)
Age in years				
18–29	10	101	09	120 (31.25)
30–49	17	126	41	184 (47.92)
>49	08	61	11	80 (20.83)
Religion				
Orthodox Christian	20	224	45	289 (75.26)
Muslim	09	34	09	52 (13.54)
Protestant	06	30	07	43 (11.20)
Residency				
Rural	27	154	50	231 (60.15)
Urban	08	134	11	153 (39.85)
Educational status				
No formal education	22	136	35	193 (50.26)
Has formal education	13	152	26	191 (49.74)
Monthly income in ETB				
< /=3000	16	139	29	184 (47.92)
>3000	19	149	32	200 (52.08)
Alcohol intake during pregnancy				
Yes	27	141	32	200 (52.08)
No	08	147	29	184 (47.92)
Caffeine intake during pregnancy				
Yes	28	156	27	211 (54.95)
No	08	131	34	173 (45.05)
Chronic illness				
Yes	23	67	16	106 (27.60)
No	12	221	45	278 (72.40)

**TABLE 2:** Distribution of reproductive health and obstetric characteristics of the respondents with their amniotic fluid volume at public hospitals in South Gondar Zone, North West Ethiopia, 2023 (*N* = 384).

Variables	Oligohydramnios	Normal AFV	Polyhydramnios	Total *N* (%)
ANC service				
Yes	24	220	45	289 (75.26)
No	11	68	16	95 (24.74)
Gravidity				
Primigravida	21	78	19	118 (30.73)
Multigravida	14	210	42	266 (69.27)
Inter‐pregnancy interval (for MG) *n* = 266				
< 2 years	26	80	30	136 (51.13)
>/=2 years	09	90	31	130 (48.87)
Hypermesis gravidarum in current pregnancy				
Yes	23	130	21	174 (45.31)
No	12	158	40	210 (54.69)
Pregnancy induced hypertension				
Yes	15	36	06	57 (14.84)
No	20	252	55	327 (85.16)
Diabetes mellitus				
Yes	06	19	11	36 (9.38)
No	29	269	50	348 (90.62)
APH in current pregnancy				
Yes	25	73	09	107 (27.86)
No	10	215	52	277 (72.14)
Estimated fetal weight				
<3000 g	21	149	19	189 (49.22)
>/=3000 g	14	139	42	195 (50.78)

**TABLE 3:** Multivariable logistic regression of maternal condition and amniotic fluid volumes in relation to other confounding variables in public hospitals south Gondar zone, Ethiopia, 2023 (*n* = 384).

Variables	Amniotic fluid abnormality		COR	AOR
	Yes	No		
Residency				
Rural	77	154	3.53 (1.36–5.87)	3.21 (1.19–5.37)
Urban	19	134	1	1
Alcohol intake				
Yes	59	141	1.66 (1.19–4.22)	1.03 (0.25–3.29)
No	37	147	1	1
Caffeine intake				
Yes	55	155	1.12 (1.06–3.97)	1.01 (0.23–2.20)
No	42	132	1	1
Chronic illness				
Yes	39	67	2.26 (2.01–4.32)	2.12 (1.33–4.61)
No	57	221	1	1
Inter‐pregnancy interval (for MG) *n* = 266				
<2 years	56	64	3.22 (2.11–5.63)	3.03 (2.18–6.28)
>/=2 two years	31	114	1	1
Hypermesis gravidarum				
Yes	62	148	1.23 (1.11–2.63)	1.19 (1.02–4.41)
No	44	130	1	1
PIH				
Yes	21	36	1.96 (1.08–3.21)	1.05 (0.73–3.39)
No	75	252	1	1
Diabetes mellitus				
Yes	17	19	3.05 (2.05–5.34)	2.16 (1.32–4.75)
No	79	269	1	1
APH				
Yes	34	73	1.62 (1.16–3.04)	1.14 (0.79–3.88)
No	62	215	1	1

## 4 DISCUSSION

The primary finding of this study is the impact of maternal condition on the status of AFV among women attending antenatal clinic in public hospitals of SGZ at their term pregnancy. The prevalence of abnormal amniotic fluid (both oligohydramnios and Polyhydramnios) in our study was 25.01% CI (19.34–28.31%). Among those, 63.54% of them had Polyhydramnios and the remaining 36.46% have oligohydramnios type of amniotic fluid abnormality. Our study finding of abnormal AFV (was 25.01%) is higher than a study done in Saudi Arabia (14.5%) (Bakhsh et al., 2021) and Spain (6.7%) (Martínez‐Frías et al., 1999). The possible reason for this much discrepancy could be the difference in study population, the difference in status of health care service, difference in nutritional status and study period.

In our study, rural residency (3.21 [1.19–5.37]) of the women correlated with three‐fold increased odds of abnormal AFV. As far as our search is concerned, we were unable to find any previous study on the effect of residency on the volume of amniotic fluid value. The possible scientific justification for this association in our study could be the lack of knowledge about the nutritional recommendations during pregnancy, delay in getting health care, and preferential work load among women in rural households than for urban residents.

Our study finding also reported that having history of chronic illness (2.12 [1.33–4.61]) correlated with a 2.12 fold increase in the odds of abnormal AFV. This finding is supported by studies done in Saudi Arabia and India (Raghuwanshi & Aggarwal, 2017; Bakhsh et al., 2021). The possible reason for this association could be could be that chronic diseases such as hypertension, asthma or renal disease have direct or indirect physiological effects on feto‐placental perfusion and oxygenation.

In our study, inter pregnancy interval less than 2 years (3.03 [2.18–6.28]) also correlated with increased odds of abnormal AFV by around three times. Although there is no any previous study that show the effect of Short birth interval on AFV, the possible justification for the association might be due to the reduced maternal backup, or effects of physiologic preparation for the subsequent pregnancy and multiple lactation on subsequent deliveries.

Our study also reported Hypermesis gravidarum (HEG) (1.19 [1.02–4.41]) during the early trimesters of pregnancy correlates with 1.19 fold increased odds of Amniotic fluid abnormality. Again, as far as our search in concerned, there is no previous study about the effect of HEG on AFV. But the possible justification for the above association could be due to the implication of HEG on maternal nutritional imbalance, fetal growth, placental dysfunction disorder and electrolyte imbalance. Those conditions in turn will have an effect on maternal‐fetal and placental perfusion and finally amniotic fluid abnormality.

Our study also reported that maternal diabetes mellitus (2.16 [1.32–4.75]) diabetes mellitus correlated with 2.16 fold increased odds of abnormal AFV. Previously published materials also supported this finding (Bakhsh et al., 2021; Raghuwanshi & Aggarwal, 2017). The possible justification for this association could be due to the hyperglycemia state of the fetus and increased fetal urination. This will again contribute to increased AFV.

## 5 CONCLUSION AND RECOMMENDATION

In this study about one‐fourth (25.01% CI [19.34%–28.31%]) of pregnancies were complicated by abnormal AFV (both oligohydramnios and Polyhydramnios). rural residency, chronic illness, short inter pregnancy interval, HEG, and maternal diabetes were correlated with increased odds of Abnormal AFV. Maternal life style modifications and health maternal conditions are required to have optimal AFV. We also recommend following researchers to conduct a separate study on Polyhydramnios and oligohydramnios.

## 6 LIMITATIONS OF THE STUDY

Despite the novelty of the study we studied both oligohydramnios and Polyhydramnios together as abnormal AFV. The multi‐center nature of the study was prone to ultrasnographic measurement difference from person to person in measuring the AFV.

## AUTHOR CONTRIBUTIONS

All authors made a significant contribution to the work reported and took part in drafting, revising or critically reviewing the article; gave final approval of the version to be published; have agreed on the journal to which the article has been submitted; and agree to be accountable for all aspects of the work.

## FUNDING INFORMATION

No funding.

## CONFLICT OF INTEREST STATEMENT

The authors declare that they have no competing interests.

## CONSENT

Not applicable.

## ETHICS STATEMENT

Ethical clearance was obtained from the college health science ethical review board of Debre Tabor University. Written informed consent was obtained from each study participants.

## Data Availability

All data included in this manuscript can be accessed from the corresponding author upon request through the email address with justified reason.
